# Long-term outcomes of patients with primary intestinal follicular lymphoma managed with watch-and-wait strategy

**DOI:** 10.1038/s41598-023-32736-9

**Published:** 2023-04-11

**Authors:** Masaya Iwamuro, Takehiro Tanaka, Daisuke Ennishi, Kazuhiro Matsueda, Masao Yoshioka, Koji Miyahara, Chihiro Sakaguchi, Mamoru Nishimura, Teruya Nagahara, Tomohiko Mannami, Ryuta Takenaka, Shohei Oka, Masafumi Inoue, Hidetaka Takimoto, Tomoki Inaba, Sayo Kobayashi, Tatsuya Toyokawa, Hirofumi Tsugeno, Seiyuu Suzuki, Sachiko Sawada, Shouichi Tanaka, Takao Tsuzuki, Hiroyuki Okada

**Affiliations:** 1 Department of Gastroenterology and Hepatology, Okayama University Graduate School of Medicine, Dentistry and Pharmaceutical Sciences, 2-5-1 Shikata-cho, Kita-ku, Okayama, Okayama 700-8558 Japan; 2 Department of Pathology, Okayama University Graduate School of Medicine, Dentistry, and Pharmaceutical Sciences, 2-5-1 Shikata-cho, Kita-ku, Okayama, Okayama 700-8558 Japan; 3 Department of Hematology and Oncology, Okayama University Graduate School of Medicine, Dentistry, and Pharmaceutical Sciences, 2-5-1 Shikata-cho, Kita-ku, Okayama, Okayama 700-8558 Japan; 4 Department of Gastroenterology and Hepatology, Kurashiki Central Hospital, 1-1-1 Miwa, Kurashiki, Okayama 710-8602 Japan; 5 Department of Internal Medicine, Okayama Saiseikai General Hospital, 2-25 Kokutai-cho, Kita-ku, Okayama, Okayama 700-8511 Japan; 6 Department of Internal Medicine, Hiroshima City Hospital, 7-33 Motomachi, Naka-ku, Hiroshima, Hiroshima 730-8518 Japan; 7 Department of Endoscopy, National Hospital Organization Shikoku Cancer Center, Kou 160, Minamiumemotomachi, Matsuyama, 791-0280 Japan; 8 Department of Internal Medicine, Okayama City Hospital, 3-20-1 Kitanagase Omote-cho, Kita-ku, Okayama, Okayama 700‑8557 Japan; 9 Department of Gastroenterology, Mitoyo General Hospital, 708 Himehama, Toyohama-cho, Kan’onji, Kagawa 769-1695 Japan; 10 Department of Gastroenterology, National Hospital Organization Okayama Medical Center, 1711-1 Tamasu, Kita-ku, Okayama, Okayama 701-1192 Japan; 11 Department of Internal Medicine, Tsuyama Chuo Hospital, 1756 Kawasaki, Tsuyama, Okayama 708‑0841 Japan; 12 Department of Gastroenterology, Nippon Kokan Fukuyama Hospital, 1844 Tsunoshita, Daimon-cho, Fukuyama, Hiroshima 721-0927 Japan; 13 Department of Gastroenterology, Japanese Red Cross Okayama Hospital, 2-1-1 Aoe, Kita-ku, Okayama, Okayama 700-8607 Japan; 14 Department of Internal Medicine, Kagawa Rosai Hospital, 3-3-1 Joto-cho, Marugame, Kagawa 763‑8502 Japan; 15 Department of Gastroenterology, Kagawa Prefectural Central Hospital, 1-2-1 Asahi-machi, Takamatsu, Kagawa 760-8557 Japan; 16 Department of Internal Medicine, Fukuyama City Hospital, 5-23-1 Zao-cho, Fukuyama, Hiroshima 721-8511 Japan; 17 Department of Gastroenterology, National Hospital Organization Fukuyama Medical Center, 4-14-17 Okinogami-cho, Fukuyama, Hiroshima 720-8520 Japan; 18 Department of Gastroenterology, Okayama Rosai Hospital, 1-10 Chikkomidorimachi, Minami-ku, Okayama, Okayama 702-8055 Japan; 19 Department of Gastroenterology, Sumitomo Besshi Hospital, 3-1 Ojicho, Niihama, Ehime 792‑8543 Japan; 20 Department of Internal Medicine, St. Mary’s Hospital, 650 Nibuno, Himeji, Hyogo 670-0801 Japan; 21 Department of Gastroenterology, National Hospital Organization Iwakuni Clinical Center, 1-1-1 Atago-cho, Iwakuni, Yamaguchi 740-8510 Japan; 22 Department of Internal Medicine, Japanese Red Cross Society Himeji Hospital, 1-12-1 Shimoteno, Himeji, Hyogo 670-8540 Japan

**Keywords:** Cancer, Gastroenterology, Oncology

## Abstract

Patients with primary intestinal follicular lymphoma are often followed-up without a specific treatment, and this approach is called the “watch-and-wait approach.” However, the long-term outcomes of this patient group have not been sufficiently investigated. We enrolled patients with primary intestinal follicular lymphoma who were diagnosed before 2016 and managed with the watch-and-wait approach in 20 institutions. We retrospectively investigated the overall, disease-specific, and event-free survival rates as well as the rate of spontaneous regression. Among the 248 patients with follicular lymphoma with gastrointestinal involvement, 124 had localized disease (stage I or II_1_). We analyzed the data of 73 patients who were managed using the watch-and-wait approach. During the mean follow-up period of 8.3 years, the follicular lymphoma had spontaneously resolved in 16.4% of the patients. The 5-year and 10-year overall survival rates were 92.9% and 87.1%, respectively. With disease progression (n = 7), initiation of therapy (n = 7), and histologic transformation to aggressive lymphoma (n = 0) defined as events, the 5-year and 10-year event-free survival rates were 91.1% and 86.9%, respectively. No patient died of progressive lymphoma. Thus, both 5-year and 10-year disease-specific survival rates were 100%. In conclusion, an indolent long-term clinical course was confirmed in the patients with primary intestinal follicular lymphoma. The watch-and-wait strategy is a reasonable approach for the initial management of these patients.

## Introduction

Follicular lymphoma is one of the most common low-grade B-cell lymphomas, accounting for 15–25% of the cases of non-Hodgkin's lymphomas^1^. Most patients with follicular lymphoma present with lymph node swelling and are diagnosed at advanced stage III or IV of the Ann Arbor Classification^2^. These patients generally experience repeated recurrence even after complete response to treatment. Randomized trials conducted in the rituximab era revealed that early initiation of rituximab improved progression-free survival, but an overall survival benefit was not shown^3^. Thus, no curative therapy has been established for advanced-stage follicular lymphoma. Asymptomatic patients with advanced-stage, low-tumor burden follicular lymphoma reportedly remain asymptomatic for years even if treatment is not initiated promptly after diagnosis. Moreover, some patients experience temporal and spontaneous tumor shrinkage. Consequently, regular check-ups with deferred initial treatment, commonly known as the “watch-and-wait” or “active surveillance” approach, remains an option for asymptomatic follicular lymphoma without bulky disease or rapid lymphoma progression^4^. Conversely, radiation therapy of 24 to 30 Gy is recommended with curative intent for a small proportion of patients with localized stage I or II follicular lymphoma.

Follicular lymphoma primarily or secondarily occurs in the gastrointestinal tract^5^. The disease entity of intestinal follicular lymphoma was established in the last two decades and is now formally considered a distinct subcategory of follicular lymphomas in the classification of tumors of hematopoietic and lymphoid tissues published by the World Health Organization (WHO)^6^. Multiple white, polypoid lesions in the duodenum, which are incidentally found during esophagogastroduodenoscopy, are the typical endoscopic features of intestinal follicular lymphoma. Among the patients with follicular lymphoma with intestinal involvement, 66.7–100% have multiple follicular lymphoma lesions in the jejunum and/or ileum^7–10^. Because of the widespread involvement of the small intestine, radiotherapy is not feasible even in cases of localized stage intestinal follicular lymphoma. Although treatment strategies have not yet been standardized, the watch-and-wait approach is often preferred in clinical settings. However, because of the rarity of this disease, the long-term outcomes of patients who are managed with this approach have not been sufficiently investigated. Therefore, in the current study, we analyzed the outcomes of 73 patients with primary intestinal follicular lymphoma who were diagnosed before 2016 and managed using the watch-and-wait approach.

## Methods

**Figure 1:**
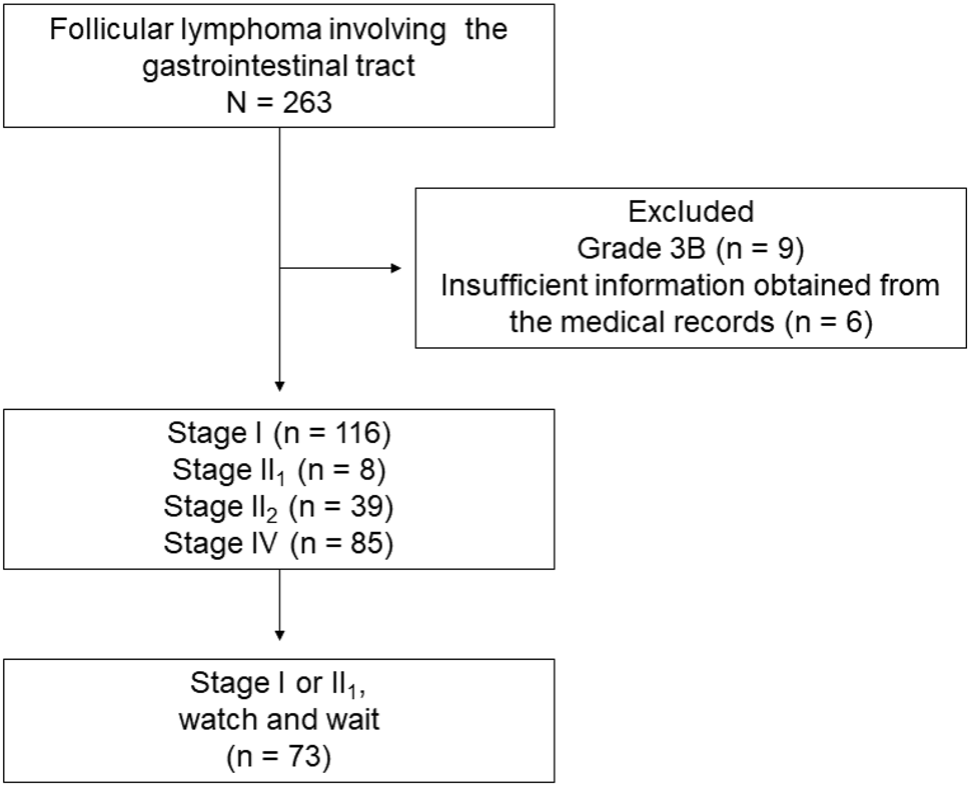
Study flow chart.

Patients with histologically diagnosed follicular lymphoma involving the gastrointestinal tract, diagnosed between July 1990 and March 2016 in Okayama University Graduate School of Medicine, Dentistry, and Pharmaceutical Sciences and 19 collaborating institutions were enrolled in this study (Fig. 1). Given our objective to investigate patients’ long-term (> 5 years) outcomes, we enrolled individuals diagnosed before 2016 in our study. Follicular lymphoma was diagnosed according to the WHO classification at that time, based on morphologic and immunophenotypic analyses of endoscopically biopsied specimens or surgically resected specimens. Histopathological grading was conducted according to the WHO criteria^6^. Gastrointestinal involvement was defined by the gross findings of endoscopic examinations, including esophagogastroduodenoscopy, colonoscopy, double-balloon enteroscopy, and/or video capsule enteroscopy. A subset of the patients examined (143/263) had also participated in our previous studies^8,11–15^.

The clinical stages of the disease were classified according to the Lugano staging system for gastrointestinal lymphoma^16^. Briefly, stage I indicates lymphoma confined to the gastrointestinal tract, where single primary, or multiple non-contiguous lesions exist. Stage II_1_ indicates lymphoma extending into the abdomen from the primary gastrointestinal site, with local nodal involvement. Stage II_2_ denotes lymphoma extending into the abdomen with distant nodal involvement, for example the mesenteric, para-aortic, para-caval, pelvic, and/or inguinal lymph nodes. Stage IV indicates disseminated extranodal involvement or gastrointestinal tract lesion with supra-diaphragmatic nodal involvement.

Of the 263 patients identified, nine were excluded from this study because they had grade 3B follicular lymphoma, which is typically managed as aggressive lymphoma (Fig. 1)^6,17^. Six patients were further excluded from this study because of insufficient information in their medical records. As described previously, we focused on patients with primary intestinal follicular lymphoma, i.e., stage I or II_1_, who were managed with the watch-and-wait strategy. Data regarding the endoscopic, radiological, biological, and pathological examinations performed were retrospectively reviewed from their clinical records to determine clinical characteristics and outcomes of the patients.

Overall survival was defined as the time from the diagnosis of follicular lymphoma to death from any cause. Disease-specific survival was defined as the time from diagnosis to death from follicular lymphoma or from the transformation of follicular lymphoma to an aggressive lymphoma. Event-free survival was defined as the time from the diagnosis of follicular lymphoma to disease progression, histologic transformation to a clinically aggressive lymphoma, initiation of therapy, or death from follicular lymphoma.

This study was approved by the ethics committees of Okayama University Hospital and of the participating institutions. The requirement for written informed consent was waived by the ethics committees of Okayama University Hospital and of the participating institutions because of the retrospective nature of the study and the analysis used anonymous clinical data. All investigations were performed in accordance with relevant guidelines and regulations and were conducted in accordance with the Declaration of Helsinki. Kaplan–Meier curves were generated using JMP (version 14.0.0; SAS Institute Inc., Cary, NC, USA). Numerical values are presented as mean ± standard deviation.

## Results

Overall, 248 patients with gastrointestinal involvement of follicular lymphoma were recruited from 20 institutions (Fig. 1). Among them, 116 patients had stage I disease, 8 had stage II_1_ disease, 39 had stage II_2_ disease, and 85 had stage IV disease. Thus, 124 patients had localized stage (stage I or II_1_) follicular lymphoma. There were no predetermined criteria for the allocation of patients into the watchful waiting or treatment intervention cohorts, and the decision regarding the treatment regimen was at the attending physician’s discretion. Patients with a localized stage (stage I or II_1_) follicular lymphoma were initially treated with radiotherapy (n = 10); rituximab monotherapy (n = 14); rituximab plus cyclophosphamide, doxorubicin, vincristine, and prednisone (CHOP, n = 12); rituximab plus pirarubicin, cyclophosphamide, vincristine, and prednisolone (n = 2); rituximab plus cyclophosphamide, vincristine, and prednisone (n = 2); surgical resection (n = 6); endoscopic resection (n = 2); surgical resection followed by chemotherapy with rituximab plus cyclophosphamide, doxorubicin, vincristine, and prednisone (n = 2); or rituximab monotherapy followed by radiotherapy (n = 1). The remaining 73 patients (58.9%) were followed-up without any specific lymphoma treatment, i.e., watch-and-wait approach. Therefore, 73 patients were analyzed.

The characteristics of the 73 patients (37 men and 36 women) with primary intestinal follicular lymphoma (stage I, n = 72; II_1_, n = 1), who were managed with the watch-and-wait strategy, are summarized in Table 1. The mean age at the diagnosis of intestinal follicular lymphoma was 65.8 years (range, 38–90 years). All patients underwent computed tomography and/or positron emission tomography during the initial staging evaluation. The patients underwent esophagogastroduodenoscopy (n = 73), colonoscopy (n = 57), video capsule enteroscopy (n = 24), and/or balloon-assisted enteroscopy (n = 7) for the assessment of gastrointestinal involvement. The duodenum was the most frequently affected organ (n = 69, 94.5%), followed by the jejunum (n = 11, 15.1%), ileum (n = 4, 5.5%), rectum (n = 2, 2.7%), stomach (n = 1, 1.4%), and colon (n = 1, 1.4%) (Suppl. Figure 1). None of the patients had lymphoma in the esophagus or cecum. Bone marrow examination was performed in 34 patients (46.6%), and none of the patients with lymphoma had bone marrow involvement.

**Table 1:** Clinical characteristics of the study patients.

	n	%
Sex		
Male	37	50.7
Female	36	49.3
Mean age at the FL diagnosis (range), years	65.8 ± 11.6 (38–90)	
Gastrointestinal involvement		
Esophagus	0	0.0
Stomach	1	1.4
Duodenum	69	94.5
Jejunum	11	15.1
Ileum	4	5.5
Cecum	0	0.0
Colon	1	1.4
Rectum	2	2.7
Stage		
Lugano I	72	98.6
Lugano II_1_	1	1.4
Endoscopy examinations performed		
Esophagogastroduodenoscopy	73	100
Colonoscopy	57	78.1
Video capsule enteroscopy	24	32.9
Balloon-assisted enteroscopy	7	9.6
Mean follow-up period (range), years	8.3 ± 4.3 (0.3–22.8)	
Median follow-up period, years	8.1	
Outcome at the last visit		
Alive with lymphoma	46	63.0
Alive, NED without treatment	12	16.4
Alive, NED with treatment	5	6.8
Alive with lymphoma after treatment	1	1.4
Dead by other causes than lymphoma	9	12.3

*FL* follicular lymphoma; *NED* no evidence of disease.

**Figure 2:**
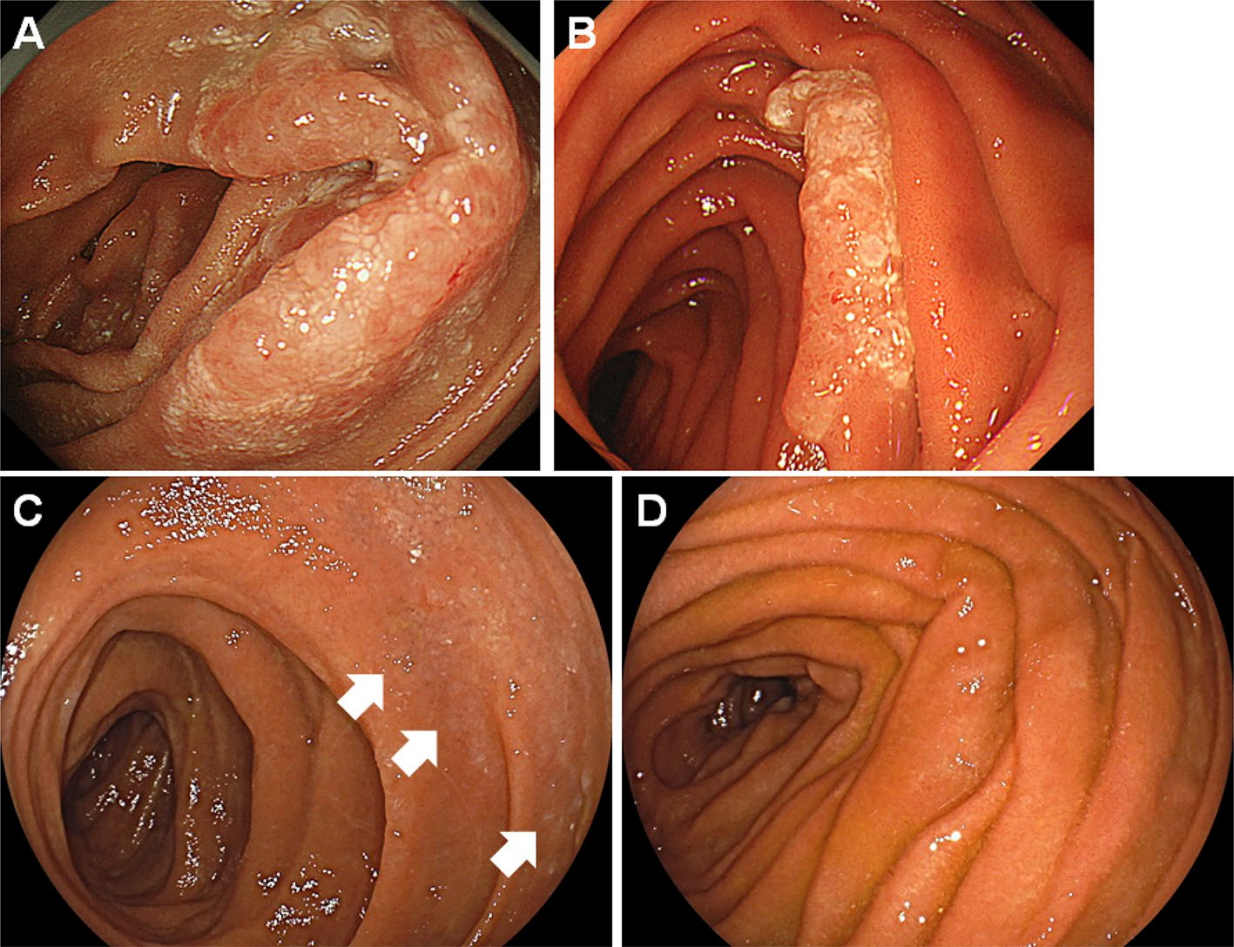
Representative endoscopic images of a case showing spontaneous regression of duodenal follicular lymphoma. A duodenal lesion showing whitish villi was detected in a 63-year-old woman (**A**). The area of the whitish lesion reduced 4 years after the diagnosis (**B**). The whitish lesion became faint 6 years after the diagnosis (**C**). The duodenal lesion disappeared and no lymphoma cells were detected on biopsied specimen 7 years after the diagnosis (**D**).

During the follow-up period, 56 patients (76.7%) underwent annual esophagogastroduodenoscopy, and 44 (60.3%) underwent annual computed tomography. Notably, 37 patients (50.7%) were followed up annually using esophagogastroduodenoscopy and computed tomography. The mean follow-up period was 8.3 years (range, 0.3–22.8 years), and the median follow-up period was 8.1 years. At the latest visit, 46 patients were alive with follicular lymphoma. The lymphoma had spontaneously disappeared in 12 patients (Fig. 2). In these patients, evaluation of the endoscopic and pathological findings of the biopsied specimen revealed the disappearance of the duodenal (n = 11) or ileal lesions (n = 1). The median duration between initial diagnosis and histological resolution was 2.6 years (range: 0.5–7.8 years). However, although six of the 11 patients with follicular lymphoma of the duodenum exhibited small intestine involvement (jejunum in four patients and ileum in two), none underwent enteroscopy during the follow-up period to confirm regression of the small intestinal lesions. After treatment, five patients were alive without follicular lymphoma and one was alive with lymphoma. Nine patients died from causes other than lymphoma, including pneumonia (n = 2), lung cancer (n = 2), gingival cancer (n = 1), gastric cancer (n = 1), pancreatic cancer (n = 1), ovarian cancer (n = 1), and chronic myelomonocytic leukemia (n = 1). None of the patients died of progressive lymphoma.

**Figure 3:**
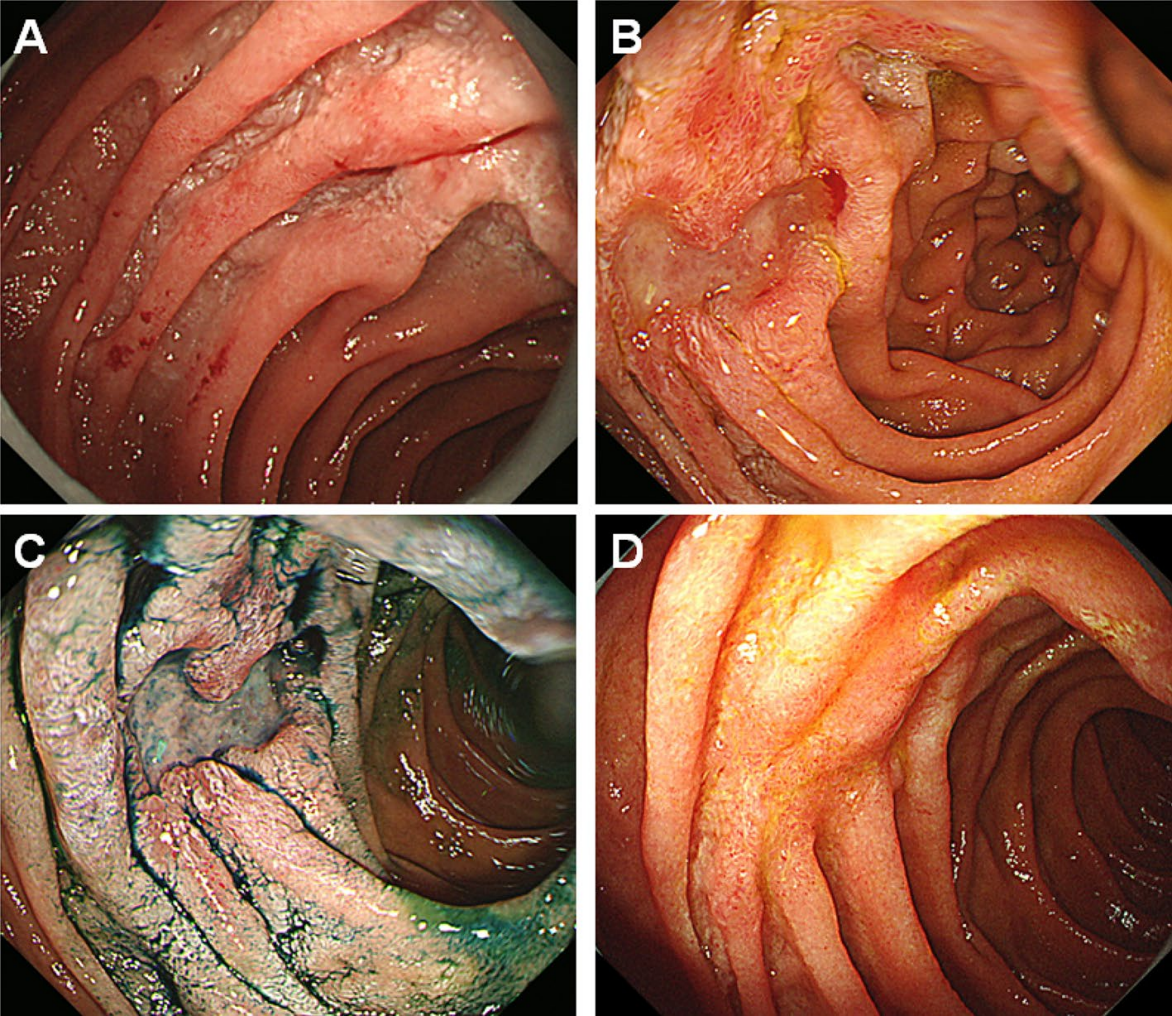
Endoscopy images of Case 3. A 64-year-old Japanese man was diagnosed with duodenal follicular lymphoma (**A**). The duodenal lesions were found to be ulcerative 2 years after the diagnosis (**B**,**C**) and disappeared after treatment with rituximab and bendamustine (**D**).

Table 2 shows the data of patients who experienced disease progression, histologic transformation, and/or initiation of treatment. Progression of follicular lymphoma occurred in six patients 0.9–7.1 years after the diagnosis. Despite progression, two patients (Case 2 and 6) were followed-up without treatment because they were asymptomatic. One patient (Case 7) underwent radiotherapy after the emergence of mesenteric lymphadenopathy. Systemic treatment was initiated in the other four patients who showed progression. In one patient (Case 3), the duodenal follicular lymphoma lesions (Fig. 3A) were identified as ulcerative 2 years after the diagnosis (Fig. 3B,C); the lesions disappeared after rituximab and bendamustine therapy (Fig. 3D). Another patient (Case 5) was prescribed rituximab monotherapy 2.7 years after diagnosis at her request. Transformation to aggressive lymphoma was observed in none of the patients.

**Table 2:** Patients who experienced disease progression or initiation of therapy.

No	Sex	Age at FL diagnosis	Involved GI tract	Stage	Events	Time to events (years)	Outcome	Follow-up period (years)
1	M	62	Duodenum, jejunum, rectum	I	Perigastric LN swelling	0.9	Alive with lymphoma after treatment	10.2
					Initiation of rituximab monotherapy	1.1		
2	M	70	Duodenum	I	Mesenteric LN swelling	1.2	Alive with lymphoma	9.5
3	M	64	Duodenum	I	Initiation of rituximab and bendamustine therapy	2.0	Alive, NED with treatment	5.8
4	F	41	Duodenum, stomach, rectum	I	Inguinal LN swelling	2.0	Alive, NED with treatment	22.8
					Initiation of rituximab monotherapy	3.5		
5	F	40	Duodenum	I	Initiation of rituximab monotherapy	2.7	Alive, NED with treatment	12.5
6	M	57	Jejunum	I	Multiple intraabdominal LN swelling	3.1	Alive with lymphoma	10.6
7	F	60	Duodenum	I	Mesenteric LN swelling	5.4	Alive, NED with treatment	8.7
					Initiation of radiotherapy	5.8		
8	F	75	Duodenum	I	Cervical and mesenteric LN swelling	7.1	Alive, NED with treatment	8.9
					Initiation of obinutuzumab and bendamustine therapy	7.5		

*FL* follicular lymphoma; *GI* gastrointestinal tract; *LN* lymph nodes; *NED* no evidence of disease.

**Figure 4:**
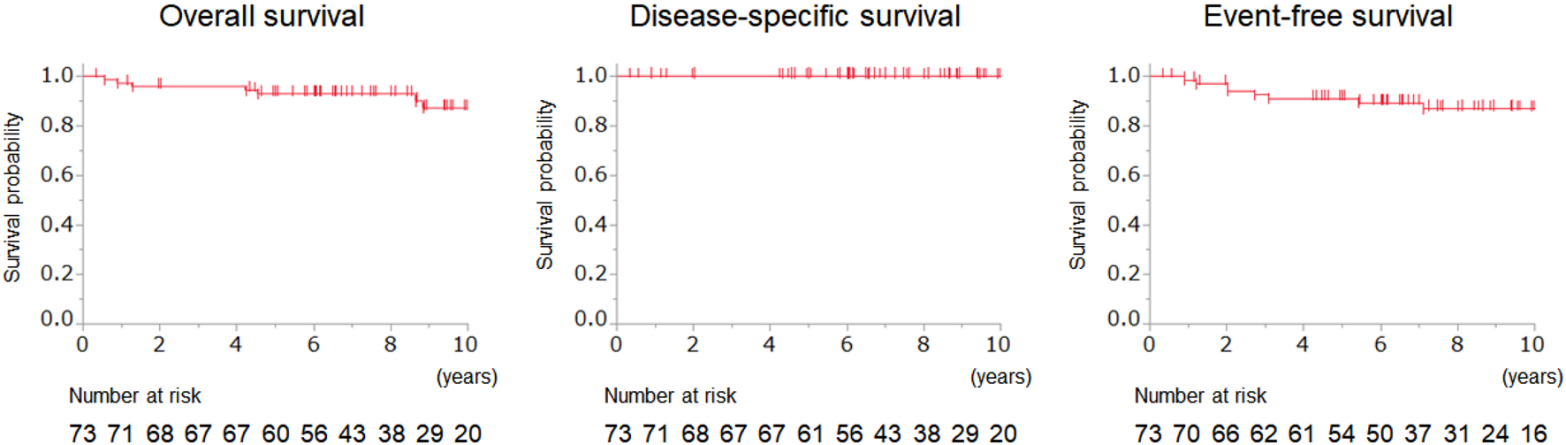
Overall, disease-specific, and event-free survival curves.

Figure 4 shows the overall, disease-specific, and event-free survival curves. The 5-year overall survival rate was 92.9% and the 10-year overall survival rate was 87.1%. As all patients died from causes other than follicular lymphoma, the 5-year and 10-year disease-specific survival rates were 100%. The 5-year and 10-year event-free survival rates were 91.1% and 86.9%, respectively.

## Discussion

Our retrospective analysis of 73 patients with primary intestinal follicular lymphoma showed that although disease progression occurred and treatment initiation was required in some patients, none died within the mean follow-up period of 8.3 years. Spontaneous regression of intestinal follicular lymphoma was observed in 16.4% of the patients. The 5- and 10-year overall survival rates were 92.9% and 87.1%, respectively. In addition, the 5- and 10-year event-free survival rates were 91.1% and 86.9%, respectively. To the best of our knowledge, this study is the first to reveal the overall, disease-specific, and event-free survival at 10 years. This study also includes the largest number of patients with primary intestinal follicular lymphoma who were managed using the watch-and-wait approach.

In 2011, Schmatz et al. conducted a retrospective study comprising 63 patients with stage I follicular lymphoma in the duodenum^18^. Of them, 24 patients received no treatment (watch-and-wait approach). During the median follow-up period of 4.6 years (55 months), seven patients (29.2%) showed spontaneous regression and 17 had stable disease, while nodal dissemination occurred in two patients (8.3%) every 5 years after diagnosis. One patient who developed nodal disease was treated with rituximab plus CHOP followed by radiotherapy, while the other patient received rituximab and bendamustine. Transformation to aggressive B-cell lymphoma did not occur, and no patient died of lymphoma. Takata et al. retrospectively investigated 125 patients with stage I and II_1_ intestinal follicular lymphoma and the participants partially overlapped with those in the present study^19^. The median follow-up period was 3.3 years (40 months). Thirty-three patients were followed using the watch-and-wait strategy; two patients (6.1%) experienced lymphoma progression. None of the patients died of lymphoma. The 5-year overall survival rate of the sample (125 patients) was 100%, and the 5-year progression-free survival rate was 93%. A prospective observational study on intestinal follicular lymphoma was recently conducted by Matysiak-Budnik et al.^20^. Among 31 patients with stage IE (n = 23), IIE (n = 3), and IV disease (n = 5), 22 patients with stage IE disease were managed with the watch-and-wait strategy. Nine patients (40.9%) achieved spontaneous remission after a median period of 5 years. Eleven patients had stable disease, one patient (4.5%) showed disease progression after 1 year of chemotherapy, and the remaining patient was lost to follow-up. Tari et al. compared patients with intestinal follicular lymphoma who were managed with the watch-and-wait approach (n = 15) or with rituximab-combined chemotherapy followed by maintenance rituximab monotherapy (n = 14)^21^. No patient in the watch-and-wait approach group had spontaneous regression in a median time of 5.3 years (63 months). Although nodal dissemination was observed in three patients (20.0%) at 13, 14, and 36 months after diagnosis, no treatment was initiated. Overall, as per the findings of previous studies, the watch-and-wait approach resulted in spontaneous regression in 0–40.9% of the patients, stage progression in 4.5–20.0%, and lymphoma-related mortality in 0%.

As mentioned above, the disease entity of primary intestinal follicular lymphoma was established within the last two decades and is now termed as duodenal-type follicular lymphoma, since it commonly involves the second portion of the duodenum^6,22^. Compared with nodal follicular lymphoma, duodenal-type follicular lymphoma is characterized by tumors of low histological grade, low risk of progression to nodal disease, and rare occurrence of large cell transformation, resulting in excellent outcomes^6^. Ongoing hypermutations and selective usage of immunoglobulin heavy-chain gene segments in duodenal-type follicular lymphoma imply an underlying mechanism triggered by antigenic stimulation. The likelihood of confined mucosal localization may be attributed to the site of initial antigenic exposure and the expression of characteristic homing receptors^6^. The long-term, estimated outcomes presented in our study are concordant with those of other studies, and validate the indolent clinical course of these patients even without treatment initiation upon diagnosis.

The methods and schedule of examinations during the follow-up period for patients with primary intestinal follicular lymphoma have not been established. Generally, periodic radiological examinations using computed tomography scanning are required to survey nodal involvement, in addition to endoscopy examinations, because nodal dissemination is the leading cause of treatment initiation. Because this was a retrospective observational study that involved multiple institutions, the initial evaluation and follow-up were performed differently between patients. However, approximately half of the patients were followed up annually using esophagogastroduodenoscopy and computed tomography. Although we consider that surveillance involving radiological and endoscopy examinations every 6 months for 1 year and annually thereafter is a reasonable and practical strategy, this aspect should be investigated further.

This study had several limitations. First, the decision to initiate treatment was made by the physician, and the included patients had been treated at various institutions by different physicians. As different institutions have different treatment policies, this study may have a participant selection bias. Particularly, seven of the eight patients diagnosed with stage II_1_ disease underwent treatment via resection, rituximab administration, and/or chemotherapy, while only one patient was assigned to a watch-and-wait policy. This may have led to an overestimation of favorable outcomes. Second, not all patients underwent endoscopic examination of the entire gastrointestinal tract. Particularly, the small intestine was not evaluated in 47 patients, although the jejunum and ileum are frequently involved^7–10^. This is because of the unavailability of video capsule or balloon-assisted enteroscopy at some institutions or because the patients had been diagnosed before the invention of the enteroscopy devices. Consequently, the prevalence of small intestinal involvement might have been underestimated. Conversely, we believe that our data reflect the real-world practice and can be applied to daily clinical settings. Third, bone marrow examination was not performed in more than half of the patients (n = 39, 53.4%), which may have resulted in an overestimation of the number of patients with localized stage (stage I or II_1_) follicular lymphoma. Fourth, variations in the timing and modalities of follow-up examinations among the patients may have influenced the analysis of the period until the event occurred. Fifth, we included patients who were diagnosed before 2016; therefore, not all patients were followed up for > 7 years.

In conclusion, we analyzed the long-term outcomes of 73 patients with primary intestinal follicular lymphoma who were managed using the watch-and-wait approach. The results of the present study indicate that the watch-and-wait approach, as proposed by the general consensus statement on nodal follicular lymphomas, is a reasonable initial management strategy for patients with primary intestinal follicular lymphoma. We hope that our results will help physicians to explain the probable course and outcome of this disease to their patients.

## Supplementary Information

Supplementary Figure S1.

## Data Availability

The datasets generated and/or analyzed during the current study are available from the corresponding author on reasonable request.

## Abbreviations

CHOP: Cyclophosphamide, doxorubicin, vincristine, and prednisone
WHO: World Health Organization
