# Supplementary material for: Long-term outcomes of patients with primary intestinal follicular lymphoma managed with watch-and-wait strategy
Source: Sci Rep. 2023 Apr 11;13:5858. doi: 10.1038/s41598-023-32736-9 (PMC10090188; doi:10.1038/s41598-023-32736-9)
Supplement: Supplementary file 1 — Supplementary Figure S1. [file 41598_2023_32736_MOESM1_ESM.docx]

Supplementary Material

Figure S1.


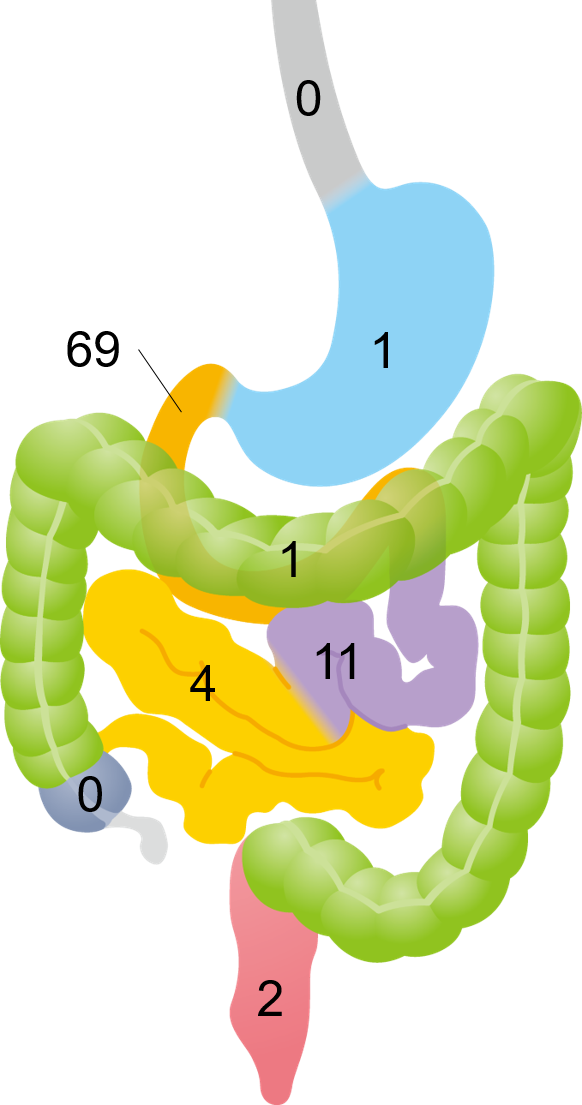


Figure S1. Schematic illustration showing the organs affected by follicular lymphoma
